# Successful treatment of macrolide-resistant *Mycobacterium abscessus* infection using multi-drug regimens including dual β-lactams and phage therapy: case reports in two children

**DOI:** 10.1128/asmcr.00087-24

**Published:** 2025-06-12

**Authors:** Masako Shimamura, Bradford Becken, Jessica Tansmore, Madison Cristinziano, Lawrence Abad, Rebekah M. Dedrick, Deborah Jacobs-Sera, Graham F. Hatfull, Robert A. Bonomo, Khalid M. Dousa

**Affiliations:** 1 Division of Pediatric Infectious Diseases, Department of Pediatrics, Nationwide Children’s Hospital and The Ohio State University 2647, Columbus, Ohio, USA; 2 Division of Infectious Diseases, Department of Pediatrics, University of Nebraska Medical Center 12284, Omaha, Nebraska, USA; 3 Clinical Pharmacy, Nationwide Children’s Hospital, Columbus, Ohio, USA; 4 Department of Biological Sciences, University of Pittsburgh, Pittsburgh, Pennsylvania, USA; 5 Department of Internal Medicine and Infectious Diseases, Louis Stokes Cleveland VA Medical Center, Case Western Reserve University 2546, Cleveland, Ohio, USA; 6 Department of Internal Medicine, Case Western Reserve University-Louis Stokes Cleveland VA Medical Center, Center for Antimicrobial Resistance and Epidemiology (Case VA CARES) 2546, Cleveland, Ohio, USA; Pattern Bioscience, Austin, Texas, USA

**Keywords:** case report, pediatric, *Mycobacterium abscessus* infection, beta-lactams

## Abstract

**Background:**

*Mycobacterium abscessus* (*M. abscessus*) infections are poorly responsive to antibiotics, with lower rates of clinical and microbiological clearance for macrolide-resistant isolates compared to macrolide-susceptible strains. Successful treatment of macrolide-resistant *M. abscessus* infection has not been reported for children.

**Case Summary:**

Here we report two children with macrolide-resistant *M. abscessus* infections who achieved clinical remission and microbiologic clearance using multi-drug treatment regimens including clofazimine, bedaquiline, linezolid or eravacycline, dual β-lactam therapy with meropenem and amoxicillin, and, in one case, phage therapy.

**Conclusion:**

Successful treatment of macrolide-resistant *M. abscessus* infections in children can be achieved using multi-drug regimens including novel dual β-lactam combinations and phage therapy. Dose adjustment with therapeutic drug monitoring can ameliorate some drug-induced toxicities in children.

## INTRODUCTION

The non-tuberculous mycobacterial (NTM) species *Mycobacterium abscessus* (*M. abscessus*) can cause pulmonary, otomastoid, and catheter-associated infections in healthy individuals as well as localized or disseminated disease in immunocompromised patients. *Mycobacterium abscessus* requires treatment with three to four drug combinations for prolonged duration (1–3). Macrolide-containing regimens have better treatment outcomes than non-macrolide-containing combinations, with 54–76% of macrolide-susceptible isolates attaining negative sputum cultures compared to 35–42% of macrolide-resistant infections (4–6). The erm(41) gene, present in *M. abscessus* subspecies *abscessus* and *bolletii*, confers inducible macrolide resistance, whereas an erm(41) mutation in *M. abscessus* subspecies *massiliense* renders this subspecies susceptible to macrolides. Optimal antibiotic combinations for macrolide-resistant *M. abscessus* infections are not established.

Risk factors for *M. abscessus* infections in children are similar to those for adults, including cystic fibrosis (CF) and other chronic lung diseases with bronchiectasis; tympanostomy tubes; central venous catheters; and immunodeficiencies, including Mendelian susceptibility to mycobacterial infection (7). Among children with CF, *M. abscessus* infection is the most common NTM infection, with a peak prevalence of 5.8% at age 11–15 years (8). NTM infections are associated with more rapid decline in lung function, morbidity, and mortality in children with CF (1). Sporadic *M. abscessus* skin and soft tissue infections can arise after trauma, surgery, needle injection, catheter insertion, or after exposure to contaminated pond or pool water (7, 9, 10). Outbreaks of *M. abscessus* pediatric dental infections have been reported after exposure to contaminated tap water during pulpotomy (11, 12). All of these pediatric cases were treated with macrolide-containing regimens. Management recommendations are based on expert opinion and are limited by a lack of pediatric formulations, dosing, and monitoring data (7). Some drugs, such as tetracyclines, have relative contraindications against long-term pediatric use. Clofazimine and bedaquiline are second-line enteral agents that have been used in pediatric *M. abscessus* infections, but pediatric formulations are not available and pediatric dosing is not well established (13, 14). Successful treatment of macrolide-resistant *M. abscessus* has not been reported in children.

Given these impediments to treatment, dual β-lactam combinations have been tested against *M. abscessus* and have shown promising results, both *in vitro* and *in vivo* (15–24). Dual β-lactam therapy targets multiple cell wall enzymes through differential binding and has been shown *in vitro* to lower the carbapenem minimum inhibitory concentrations of clinical isolates into a range compatible with clinical susceptibility (16). Successful *M. abscessus* treatment using dual β-lactams in combination with other anti-mycobacterial drugs has been reported for several adults (18–20, 22, 24), including macrolide-resistant isolates, and one child who had a macrolide-susceptible isolate (21). Here we report two pediatric cases with macrolide-resistant *M. abscessus* infections that were successfully treated with multi-drug regimens including dual β-lactams. The Institutional Review Boards at the University of Nebraska Medical Center and Nationwide Children’s Hospital designated these case reports as “not human subjects research,” not requiring informed consent. For Case 1, institutional Health Insurance Portability and Accountability Act (HIPAA) authorization was not required; for Case 2, the guardian provided HIPAA authorization to the author (M.S.) for this case report.

## CASE PRESENTATION

### Case 1

A 4-year-old male returned to Pediatric Infectious Diseases having recently completed treatment for bilateral mastoiditis caused by macrolide-resistant *M. abscessus* subspecies *abscessus*. His symptoms improved and inflammatory markers normalized (erythrocyte sedimentation rate [ESR] <15 mm/h, c-reactive protein [CRP] <0.7 mg/dL) quickly while on tigecycline (2 mg/kg IV q12h, discontinued at month five due to diarrhea and transaminitis: AST 324 (reference range, 10–45 U/L) and ALT 231 (reference range, 12–45 U/L)), parenteral amikacin (20 mg/kg IV qday), and linezolid (10 mg/kg PO daily). Treatment was discontinued at 6 months rather than a conventional 12-month course due to the early clinical response and drug toxicity to tigecycline at month 5. However, symptoms relapsed less than 2 weeks after completing therapy. Repeat acid-fast bacillus (AFB) cultures were again positive for macrolide-resistant *M. abscessus* (Table 1). Treatment was restarted with parenteral amikacin, oral linezolid, and oral doxycycline (2.2 mg/kg IV q12h), with doxycycline transitioned to eravacycline (1.5 mg/kg IV daily) due to family preference, given difficulties in administering oral medications. Bedaquiline (loading dose 160 mg PO daily, then 80 mg PO three times a week transitioning to 60 mg PO three times a week) was added after insurance approval. Clofazimine (50 mg PO three times a week, 2.5 mg/kg/dose, or 1 mg/kg/day) was also added after single-patient Investigational New Drug (spIND) authorization was obtained. The patient’s right ear otorrhea again resolved quickly, but he continued to have left-sided otorrhea, as well as bilateral hearing loss (L > R). After symptom resolution in the right ear, he underwent uncomplicated placement of a right cochlear implant that subsequently remained free of infection. The patient eventually underwent complex mastoidectomy and left-sided cochlear implant, but the implant was complicated by wound dehiscence due to *M. abscessus* infection and was eventually lost. A wire was left in place to stent the auditory nerve despite concern that this wire could be a nidus for persistent infection, since removal of the wire was felt to preclude any future cochlear implant on the left side.

**TABLE 1:** Antimicrobial susceptibility testing results for *M. abscessus* isolates^*c*^

Antibiotic	Case 1				Case 2	
	First isolate		Second isolate			
	MIC, μg/mL	Interpretation^*a*^	MIC, μg/mL	Interpretation^*a*^	MIC, μg/mL	Interpretation^*a*^
Amikacin	16	S	4	S	>256	R
Bedaquiline	ND	ND	0.06	NI	0.12	NI
Cefoxitin	64	I	32	I	32	I
Ciprofloxacin	>4	R	>4	R	>4	R
Clarithromycin	8	R	>16	R	>16	R
Clofazimine	ND	ND	0.12	NI	0.25	NI
Doxycycline	>16	R	1	S	>8	R
Eravacycline	ND	ND	100% inhibition^*b*^	NI	0.12	NI
Ertapenem	ND	ND	ND	ND	>128	NI
Imipenem	ND	ND	>32	R	8	I
Linezolid	4	S	≤1	S	16	I
Meropenem	ND	ND	>64	R	8	I
Moxifloxacin	8	R	>4	R	>4	R
Omadacycline	ND	ND	100% inhibition^*b*^	NI	0.5	NI
Tedizolid	ND	ND	ND	ND	1	NI
Tigecycline	ND	ND	0.25	NI	0.25	NI
Trimethoprim- sulfamethoxazole	>8/152	R	4/76	R	>4/76	R

^*a*^ Susceptibility testing interpretation according to cutoffs using Clinical & Laboratory Standards Institute (CLSI)-recommended broth microdilution minimum inhibitory concentration (MIC) method for non-tuberculous mycobacteria.

^*b*^ There are no CLSI breakpoints or interpretative criteria to indicate MIC by percentage growth inhibition, so “100% inhibition” is the reported interpretation according to the reference laboratory (ARUP Laboratories).

^*c*^ MIC, minimum inhibitory concentration; S, susceptible; I, intermediate; R, resistant; NI, no CLSI interpretation available; ND, not done.

**Fig 1:**
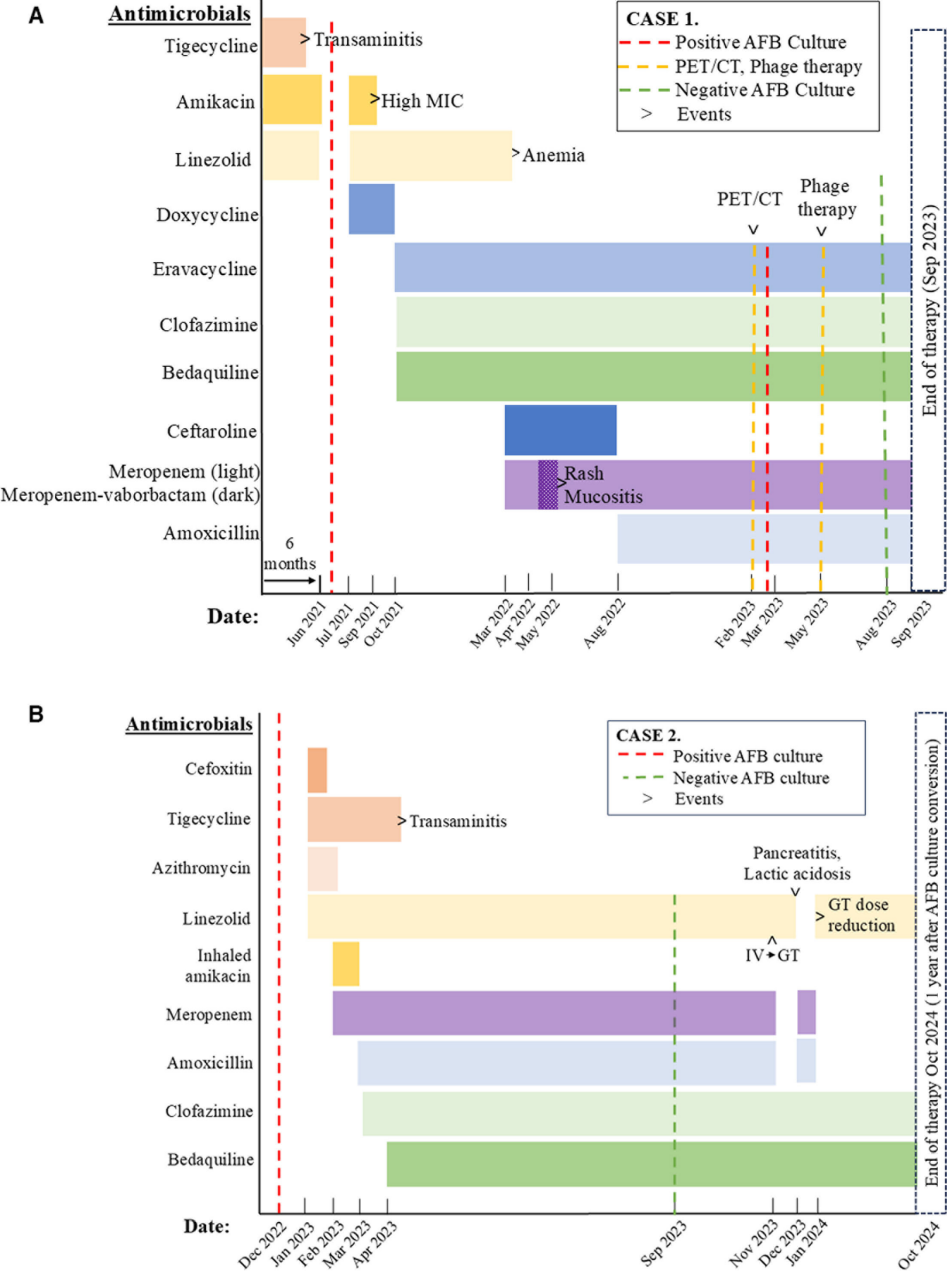
Treatment timelines. Antibiotics are shown as bars. Positive cultures are shown with red lines. PET/CT and phage therapy are shown with gold lines. Microbiological clearance is shown with green line. Events and toxicities are denoted on timelines. (**A**) Case 1. (**B**) Case 2. AFB, acid fast bacillus; IV, intravenous; GT, gastro-jejunostomy tube; MIC, minimum inhibitory concentration; PET/CT, positron emission tomography/computed tomography.

During treatment, the patient developed symptomatic anemia (hemoglobin 6.4 g/dL; reference range, 9.5–14 g/dL) requiring transfusion, and linezolid was discontinued. Amikacin was also discontinued due to the isolate’s high minimum inhibitory concentration (32 µg/mL; susceptible, ≤16 µg/mL) and concern for ototoxicity. Owing to limited antibiotic options, the isolate was sent to a research laboratory at Cleveland VA Medical Center for β-lactam synergy testing and to the University of Pittsburgh for phage susceptibility testing, although the strain has a smooth colony morphotype and was not susceptible to any of the phages tested. While awaiting these results, empiric dual β-lactam therapy with ceftaroline (10 mg/kg IV q8h) and meropenem (20 mg/kg IV q8h) was started in place of amikacin and linezolid. Shortly thereafter, based on literature suggesting greater *in vitro* susceptibility of *M. abscessus* to meropenem-vaborbactam than meropenem in dual β-lactam combinations, meropenem was changed to meropenem-vaborbactam pending *in vitro* synergy testing results (25). After several weeks of treatment, the patient developed a rash with oral mucosal involvement unresponsive to antihistamines and steroids. The meropenem-vaborbactam was discontinued, and meropenem was restarted in its place. β-lactam synergy testing showed *in vitro* susceptibility to meropenem with amoxicillin (Table 2). Therefore, ceftaroline was discontinued, and amoxicillin (875 mg PO q12h) was initiated in its place in order to reduce the number of IV medications and decrease broad-spectrum antibiotic pressure on the intestinal microbiome. The final antibiotic regimen was IV meropenem and eravacycline, with oral bedaquiline, clofazimine, amoxicillin, with intermittent ciprofloxacin-dexamethasone (Ciprodex) otic drops and neomycin-polymyxin B-hydrocortisone (Cortisporin) otic drops (Fig. 1A).

**TABLE 2:** Dual β-lactam *in vitro* synergy testing

Antibiotic (µg/mL)	Test method	MIC (µg/mL)	
		Case 1	Case 2
Imipenem	Alone	8	16
Cefuroxime	Alone	16	64
Cefdinir	Alone	32	16
Ceftaroline	Alone	16	32
Meropenem	Alone	>64	16
Imipenem + cefuroxime	Cefuroxime fixed at 4 µg/mL	≤0.12	0.25
Imipenem + cefdinir	Cefdinir fixed at 4 µg/mL	≤0.12	0.25
Imipenem + ceftaroline	Ceftaroline fixed at 4 µg/mL	≤0.12	0.25
Imipenem + amoxicillin + relebactam	Amoxicillin fixed at 8 µg/mL Relebactam fixed at 4 µg/mL	ND^*a*^	≤0.12
Meropenem + amoxicillin	Amoxicillin fixed at 8 µg/mL	≤0.12	1
Meropenem-vaborbactam + amoxicillin	Amoxicillin fixed at 8 µg/mL	8	≤0.12
Meropenem + amoxicillin + relebactam	Amoxicillin fixed at 8 µg/mL Relebactam fixed at 4 µg/mL	≤0.12	ND
Meropenem-vaborbactam + cefuroxime	Cefuroxime fixed at 4 µg/mL	0.25	ND
Meropenem-vaborbactam + cefdinir	Cefdinir fixed at 4 µg/mL	0.25	ND
Meropenem-vaborbactam + ceftaroline	Ceftaroline fixed at 4 µg/mL	0.25	ND
Durlobactam + cefuroxime	1:1 µg/mL	0.12	ND
Durlobactam + sulbactam	1:1 µg/mL	32	ND
Durlobactam + sulbactam + amoxicillin	Amoxicillin fixed at 8 µg/mL	0.12	ND

^*a*^ ND, not done.

With this multi-drug combination therapy, the patient’s otorrhea resolved, inflammatory markers normalized, and the patient tolerated the therapy without further adverse effects. The infection in the right ear resolved without further complications. However, at 18 months of antibiotic therapy, a positron emission tomography/computed tomography (PET/CT) scan showed ongoing increased uptake in the left ear, concerning for persistent inflammation and infection due to the retained hardware. A new culture was obtained despite the patient showing no outward symptoms of ongoing disease. The culture grew *M. abscessus,* and the isolate was re-evaluated for phage susceptibility. This *M. abscessus* isolate was now found to have a rough colony morphotype and was susceptible to phage Muddy and its derivatives (26). During phage treatment, the infection in the left ear resolved. Follow-up AFB cultures and PET/CT prior to the end of therapy did not show any uptake indicative of an ongoing infection. The patient now has two functioning cochlear implants without signs of infection at 10 months following completion of therapy. Further details of the phage analysis and preparation will be described elsewhere.

### Case 2

**Fig 2:**
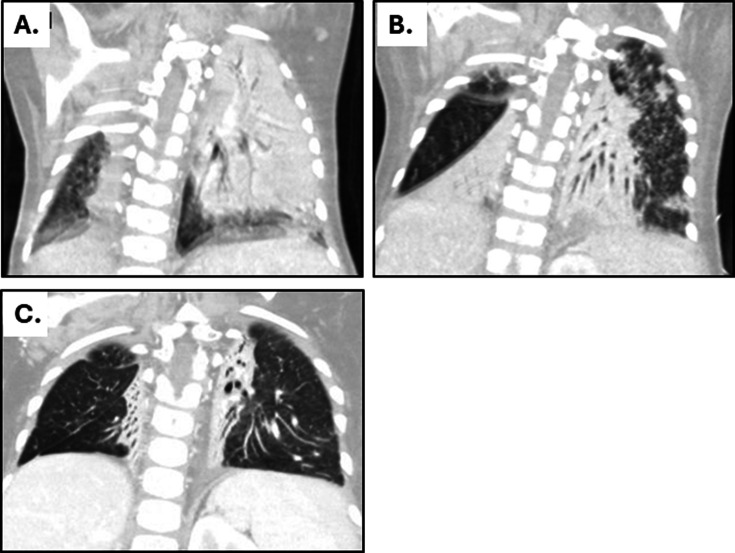
Case 2: chest CT with contrast. Images shown at (**A**) treatment initiation, (**B**) month 2 of therapy, and (**C**) month 20, end of treatment. CT images prior to the onset of *M. abscessus* infection are not available.

A male infant was born at 35 weeks’ gestational age by urgent cesarean section for poor biophysical profile. Postnatally, magnetic resonance imaging (MRI) of the head revealed C7-T1 hemorrhage and myelomalacia consistent with *in utero* spinal injury or infarct. He required a tracheostomy for ventilator support and subsequently had a small bowel resection due to volvulus, resulting in short-gut syndrome requiring continuous feeds via gastrojejunostomy tube (G-J tube, or GT). At age 7 months, he was transferred to a chronic care facility for adults and children. Starting at age 9 months, he was admitted repeatedly due to fever and respiratory distress, during which he was treated with vancomycin, meropenem, piperacillin-tazobactam, amikacin, trimethoprim-sulfamethoxazole, minocycline, and nasal mupirocin (all given at standard dosing for weight and age). At age 19 months, he was admitted to the intensive care unit due to high fevers and respiratory failure with multifocal atelectasis on CT (Fig. 2A). Multiple tracheal and bronchoalveolar lavage (BAL) cultures were positive for *M. abscessus*, identified via secA sequencing. Subspecies identification was not performed. Empiric IV cefoxitin (50 mg/kg IV q8h), tigecycline (1 mg/kg IV q12h), linezolid (10 mg/kg IV q8), and azithromycin (10 mg/kg per GT qday) were initiated, but the *M. abscessus* isolate was later found to be resistant to macrolides, aminoglycosides, quinolones, trimethoprim-sulfamethoxazole, and doxycycline (Table 1). Antibiotics were changed to linezolid (10 mg/kg IV q8h), tigecycline (1 mg/kg IV q8h), meropenem (40 mg/kg IV q8h), and inhaled amikacin (500 mg inhaled q12h) (Fig. 1B). Inhaled amikacin was given by the treating clinician in hopes of attaining antibiotic concentrations above the MIC in the airways, pending identification of an alternative antibiotic.

Given the known poor outcome of macrolide-resistant *M. abscessus* infection, the isolate was sent to a research laboratory at Cleveland VA Medical Center for *in vitro* dual β-lactam synergy testing. Dual β-lactam combinations showed *in vitro* synergy, lowering the imipenem MIC from 16 µg/mL as a single agent to 0.25 µg/mL when combined with cephalosporins. However, imipenem was relatively contraindicated for this patient due to an underlying seizure disorder, so meropenem was tested in combination with amoxicillin, which can be given orally and is well-tolerated in children. The MIC for meropenem alone was 16 µg/mL, but combined with amoxicillin (8 µg/mL), the meropenem MIC improved to 1 µg/mL (Table 2). Given this evidence of *in vitro* synergy, inhaled amikacin was discontinued on treatment week 5, and amoxicillin (50 mg/kg/day per GT, divided TID) was initiated with continued meropenem, linezolid, and tigecycline.

At week 6, clofazimine became available via spIND approval and was initiated at 50 mg per GT, twice weekly (1 mg/kg/day) (2). To administer the gelatin capsule via GT, a suspension was prepared by melting the capsule in hot water according to the method of Valinetz et al. (27). Serum clofazimine levels were 0.70 µg/mL (day 7), 0.27 µg/mL (week 5), and thereafter ranged between 0.11 and 0.43 µg/mL (tested q1–3 months) with a therapeutic goal between 0.25 and 2.0 µg/mL based on tuberculosis guidelines (28–30). On week 9, tigecycline was discontinued due to transaminitis with direct hyperbilirubinemia, and bedaquiline was initiated (60 mg per GT once daily for 2 weeks, then 60 mg, three times weekly) (Fig. 1B).

With this regimen of meropenem, amoxicillin, linezolid, clofazimine, and bedaquiline, fevers resolved, and the patient’s ventilator settings improved, but the chest CT showed persistent multifocal consolidation with diffuse miliary nodules (Fig. 2B). At 8 months of treatment, a BAL culture was negative for *M. abscessus*. Meropenem and amoxicillin were discontinued, and linezolid was changed from IV to enteral (10 mg/kg per GT, q8h), but he rapidly developed pancreatitis and lactic acidosis. Meropenem and amoxicillin were substituted for linezolid until the pancreatic enzymes and serum lactate normalized. As the literature for multi-drug-resistant tuberculosis indicates that linezolid toxicity is associated with blood trough levels > 2.0 mg/L (31, 32), enteral linezolid was restarted at a lower dose (10 mg/kg per GT, q12h), and the AUC_0-24_ was calculated on day 2 of therapy (trapezoidal method, serum concentrations collected at four time points). The AUC_0-24_ was 146 mg*h/L with a Cmin/trough of 0.74 mg/L. Weekly surveillance testing showed no recurrence of pancreatitis or lactic acidosis, and subsequent linezolid troughs were <2 mg/L. After 20 months of therapy, chest CT imaging improved (Fig. 2C), and BAL cultures were negative for 12 months, so antibiotics were discontinued. At 5 months without anti-mycobacterial therapy, the patient’s tracheal aspirate cultures remain negative for acid-fast bacilli. He has had intermittent respiratory symptoms due to viral and typical bacterial infections, which have improved without receiving further anti-mycobacterial therapy.

## DISCUSSION

Infections caused by *M. abscessus* pose formidable challenges for infectious disease providers, stemming primarily from the organism’s inherent resistance to numerous commonly employed antimicrobial agents, including anti-tuberculous medications, and its remarkable adaptability to survive in diverse environments, including the formation of biofilms (33). The therapeutic landscape for *M. abscessus* infections has become increasingly complex in recent times, with a notable surge in the incidence of refractory infections (34). Isolates carrying the *erm41* gene mutation, encoding erythromycin methylation and conferring inducible resistance to macrolides (35), have been associated with significantly poorer treatment outcomes, with some reports suggesting success rates as low as 35% (5). *Mycobacterium abscessus* infections are often described as more challenging than managing extensively drug-resistant tuberculosis. While the recently published Infectious Diseases Society of America/American Thoracic Society (IDSA/ATS) guidelines offer recommendations for therapy (3), there is no clear guidance in the case of multi-drug-resistant *M. abscessus* infections. This often leads physicians to seek the expertise of mycobacterial specialists at institutions such as National Jewish Health for guidance. These two cases serve as prime illustrations of all the aforementioned issues.

Isolates of *M. abscessus* produce β-lactamase (Bla_Mab_), known to limit the efficacy of β-lactam antimicrobials (36). When the Bla_Mab_ gene is knocked out in these isolates, MIC readings of amoxicillin become comparable to imipenem, demonstrating the ability of this enzyme to hydrolyze penicillin (37). This effect is particularly pronounced for penicillins, to some extent for cephalosporins, and to a lesser degree for carbapenems (38). Notably, amoxicillin has been found to inhibit DD-carboxypeptidase, a critical enzyme involved in peptidoglycan elongation (17). Meropenem, in contrast, targets both D,D-transpeptidases and L,D-transpeptidases (LDTs), with particular potency against LDTs. LDTs are responsible for forming the 3 → 3 cross-links that predominate in the *M. abscessus* cell wall. Carbapenems, such as meropenem, are uniquely effective at inhibiting these enzymes. The complementary inhibition of different penicillin-binding proteins by amoxicillin and meropenem provides the mechanistic rationale for using these agents in combination (16). The interest in repurposing β-lactams in combination with β-lactamase inhibitors (BLI) for the treatment of *M. abscessus* infections surged with the introduction of new-generation BLIs. For instance, the addition of relebactam to imipenem significantly reduced MICs and restored amoxicillin susceptibility (10), while durlobactam similarly has been observed to significantly improve the susceptibility of amoxicillin when used in conjunction with imipenem (17, 39). As research advanced, the concept of dual β-lactam therapy gained ground, hypothesizing that a combined β-lactam could interact with multiple LDTs, potentially achieving cell wall inhibition without the need for BLI “target redundancy’’ (15, 16). This approach has been exemplified by combinations such as ceftaroline and imipenem (15), doripenem and cefdinir (38), ceftazidime and ceftaroline (40), imipenem and cefdinir, imipenem and cefoxitin (41), sulopenem with cefuroxime (23), tebipenem, and cefuroxime combined with amoxicillin (42), casting doubt on the necessity for Bla_Mab_ inhibition. Clinicians have increasingly adopted this strategy for treating macrolide-resistant *M. abscessus* infections, with reports of notable clinical success (18–21).

In addition to drug resistance, drug toxicities frequently limit the choice and duration of antibiotics used to treat *M. abscessus* infections. For both cases, tigecycline was discontinued early in the treatment course due to hepatotoxicity. For Case 2, linezolid was an important component of the enteral regimen, but the patient developed pancreatitis and lactic acidosis when transitioned from IV to enteral formulations. Based on the literature for drug-resistant tuberculosis treatment, linezolid was restarted at lower dosing with documented serum troughs < 2 µg/mL (31, 32), without further episodes of pancreatitis or lactic acidosis. Similarly, clofazimine levels were monitored in Case 2 to demonstrate adequate absorption of the dissolved capsule formulation in the setting of short-gut syndrome. Serum clofazimine levels were detectable but intermittently fell below the range typically targeted for tuberculosis. However, microbiological clearance was achieved with this dosing, similar to another report of successful *M. abscessus* clearance among children with CF who had low serum clofazimine levels (43). Furthermore, this dosing regimen was not associated with common adverse effects such as skin discoloration or gastrointestinal symptoms. As clofazimine is highly lipophilic and accumulates in tissues, the low serum levels may not reflect tissue concentrations enabling microbiological clearance in the lungs. These cases illustrate the utility of therapeutic drug monitoring to avoid drug toxicities while still providing efficacious treatments against drug-resistant *M. abscessus* infections. Finally, for Case 1, bacteriophage therapy appeared to contribute to eradication of a hardware-associated infection.

The clinical scenarios presented in the preceding vignettes posed unique and formidable challenges for several reasons: (i) the *M. abscessus* strain carried the erm(41) gene mutation, (ii) both patients experienced significant drug toxicity, making intravenous therapy intolerable, (iii) in Case 1, the presence of metallic ear foreign body likely led to persistence of the infection due to biofilm formation. In this report, we detail cases where dual β-lactam therapy was employed as part of multi-drug regimens, ultimately leading to successful clinical resolution of the infection. However, it is important to acknowledge certain limitations as we present this case. First, while the exact role of dual β-lactam therapy in the treatment of these infections is difficult to determine, we suspect that the addition of β-lactams to the multidrug regimen played a significant role. To date, there are no published reports of *M. abscessus* treatment failure in patients receiving dual β-lactam containing regimens, although this could reflect a publication bias against cases with negative or inconclusive findings. For both cases, the combination of meropenem and amoxicillin was utilized, so the effectiveness of other dual β-lactam combinations was not determined. Second, although whole genome sequencing was not conducted, it could have provided valuable insights into the presence or activity of Bla_Mab_ in the *M. abscessus* isolate. We hypothesize that the absence of active Bla_Mab_ may explain the positive response to the combination therapy mentioned above. Lastly, while it remains uncertain whether a microbiological cure was achieved in case one, radiographic findings were reassuring following phage therapy. In contrast, case two had negative BAL cultures during treatment that confirmed microbiologic clearance.

In conclusion, the use of prolonged multi-drug combination therapy including dual β-lactams and, in one case, phage therapy, for treating macrolide-resistant *M. abscessus* in these two pediatric patients proved to be safe, effective, and well-tolerated. Nevertheless, further research is imperative to explore the ideal combination of β-Lactams, establish standardized protocols, and increase the accessibility of susceptibility testing for dual β-Lactams.

## AFTER EPUB

*[This article was published on 12 June 2025 with authors missing from the byline and with errors in the text describing Case 1. These items were corrected in the current version, posted on 30 July 2025.]*
